# Racial and Ethnic Health Inequalities in Accelerated Transitions in Mild Cognitive Impairment and Dementia

**DOI:** 10.1093/geroni/igaa057.945

**Published:** 2020-12-16

**Authors:** Ginny Natale, Yun Zhang, Sean Clouston

**Affiliations:** 1 Stony Brook University, Stony Brook, New York, United States; 2 SUNY Stony Brook University, Stony Brook, New York, United States

## Abstract

Background: It is unclear how the expected risk of Mild Cognitive Impairment (MCI) and conversion to dementia in the United States differs by Race/ethnicity and gender in older adults. It is unclear whether expectations about racial disparities have changed in light of changing trends in obesity and diabetes. Method: We analyzed six waves of the National Health and Aging Trends Study (NHATS), a nationally representative prospective cohort study of U.S. residents aged 65 and older. Participants were classified into four clinically meaningful mutually-exclusive groups: unimpaired, MCI, probable dementia and death. We used multistate survival models to examine individuals’ cognitive transitions and reported predictors using hazards ratios. Results: Participants (n=6,078) were 77 years old, on average, and the majority were white, females and high school graduates. Increased age was associated with risk of MCI and death. Adjusted for obesity status and diabetes, Hispanics were had a net reduced risk of death from dementia (HR=0.60; 95% CI [0.38-0.96]) but lived longer with MCI and dementia before death. Cognitively normal Blacks faced significantly increased risk of developing MCI (HR=2.54 95% CI [1.81-3.53]) and dementia (HR=1.77 95% CI [1.34-2.35]). Obese and overweight elderly had reduced risk across transitions to MCI then to dementia, but similar risks of death compared to lower body mass indices (BMI). BMI and diabetes reveal a suppression effect and racial inequalities became more apparent. Discussion: Results showed persistent racial inequalities in MCI, probable dementia, and death, supporting the theory that racial/ethnic factors are important to expectations of aging Americans.

